# Supplementary material for: The phased pan-genome of tetraploid European potato
Source: Nature. 2025 Apr 16;642(8067):389–97. doi: 10.1038/s41586-025-08843-0 (PMC12158759; doi:10.1038/s41586-025-08843-0)
Supplement: Supplementary file 5 — Supplementary Figs. 3–14, 20, 21, 23–27 and 34–44. [file 41586_2025_8843_MOESM5_ESM.zip › suppl_figure_23/suppl_figure_23d_chr04.pdf]

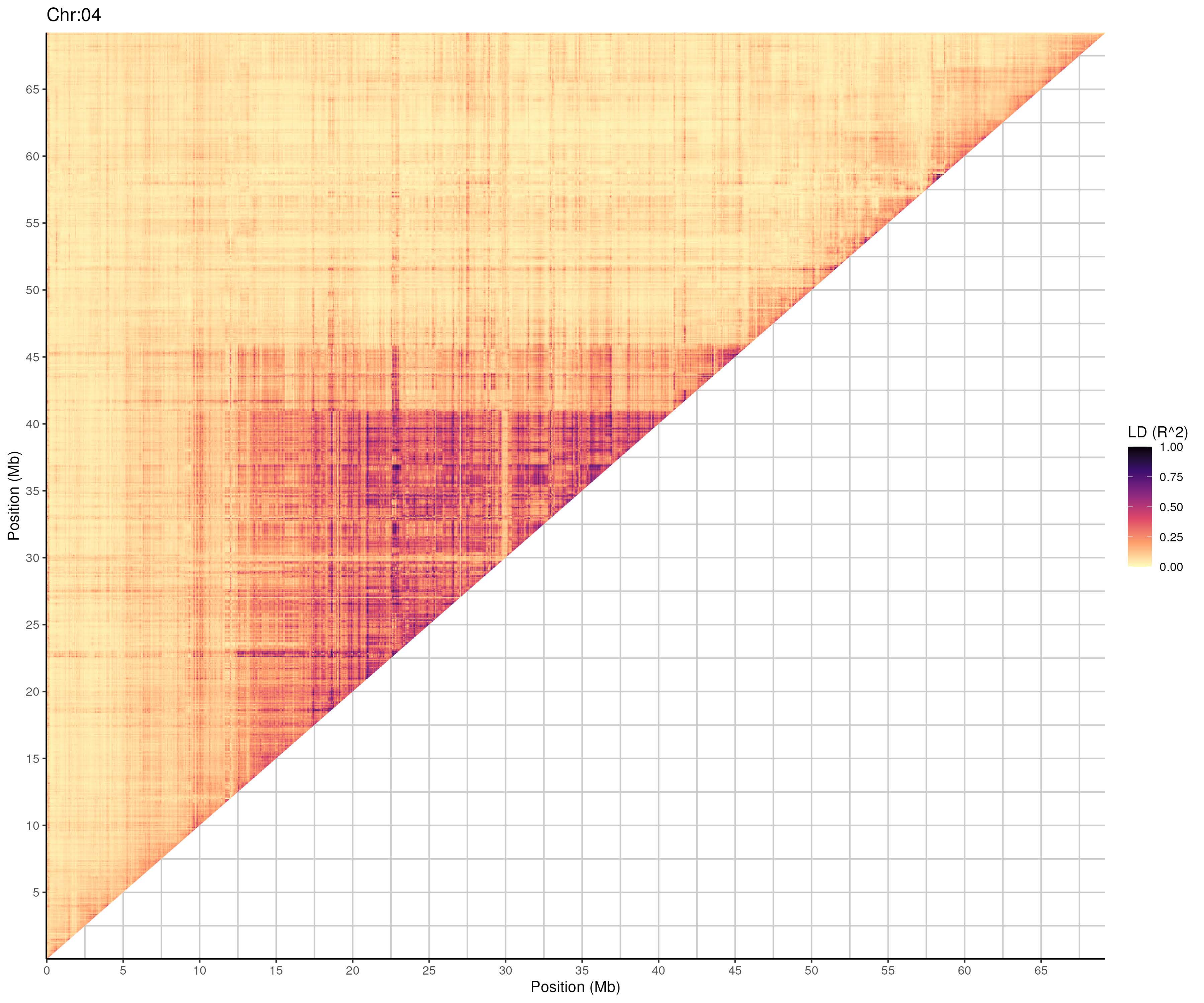
Chr:04
Position (Mb)
Position (Mb)
LD (R^2)
1.00
0.75
0.50
0.25
0.00
0 5 10 15 20 25 30 35 40 45 50 55 60 65
5 10 15 20 25 30 35 40 45 50 55 60 65
